# The Interrelatedness of Gender-Stereotypical Interest Profiles and Students’ Gender-Role Orientation, Gender, and Reasoning Abilities

**DOI:** 10.3389/fpsyg.2019.01402

**Published:** 2019-06-25

**Authors:** Lisa Ehrtmann, Ilka Wolter, Bettina Hannover

**Affiliations:** ^1^ Department of Competencies, Personality, Learning Environments, Leibniz Institute for Educational Trajectories, Bamberg, Germany; ^2^ Department of Education and Psychology, Freie Universität Berlin, Berlin, Germany

**Keywords:** vocational interest, academic interest, gender-role orientation, interest profiles, gender differences, secondary school

## Abstract

This study investigates early secondary school students’ gender-stereotypical interest profiles and how they relate to students’ gender-role orientation, i.e., their traditional or egalitarian attitudes toward gender roles. Gender-stereotypical interest profiles are described by relatively high interests in either female- or male-stereotypical domains and low interests in domains that are not associated to the own gender group. In a study conducted with 4,457 students (49.2% female, sixth graders) with data from the German National Educational Panel Study^1^, four interest profiles were derived from the combined latent profile analysis of two academic interest domains (mathematics and German) and six vocational interest domains (realistic, investigative, artistic, social, enterprising, and conventional). Aside from two gender-stereotypical interest profiles, two gender-undifferentiated interest profiles were found. One undifferentiated interest profile was marked by generally high interests in all domains, the other by generally low interests in all domains. Students in the male-stereotypical interest profile had high values in the mathematics, realistic, investigative, and enterprising domains and low interest in the German, artistic, social, and conventional domains. The female-stereotypical interest profile was marked by the opposite pattern. The results further showed that students more likely belonged to the high or female interest profiles when they expressed egalitarian gender-role orientations. Also, boys were more likely members of the female interest profile than were girls of the male interest profile. Students with low reasoning skills were generally more likely members of the low interest profile group. Results are discussed with respect to the question whether interest profiles are more predictive of students’ academic development than single domain-specific measures of interest.

## Introduction

Interests are important for academic as well as vocational decisions and an important motivational component of learning and school success (Wigfield and Cambria, 2010). Academic and vocational decisions have repeatedly been found to be highly dependent on young people’s gender (Statistisches Bundesamt [German Federal Statistical Office], 2017). This becomes a problem when considering differences in income and prestige of male- and female-dominated occupations and the uneven distribution of men and women in high-status positions (e.g., Oh and Lewis, 2011). Therefore, when investigating interests as a precursor of vocational decisions, gender differences have to be taken into account. In this study, we aim at finding gendered interest profiles in a sample of sixth grade students and investigated how students’ attitudes toward gender roles as well as their reasoning abilities were related to their probability to belong in different profiles. Going beyond previous studies which, in a variable-centered approach, investigated gender differences in particular school subject areas, we compare interest profiles that are gender congruent, gender incongruent, or are gender neutral but differ with regard to interest levels being generally low or high. We argue that employing such a person-centered perspective to examine intraindividual profiles of interests and corresponding gender differences can help to improve our understanding of gendered educational aspirations and vocational career paths (cf., Seidel, 2006; Viljaranta et al., 2009; Chow et al., 2012; Jurik et al., 2013, 2014).

Schiefele (1991) described interest as a motivational component which is content-specific and defined by an intrinsic feeling- and value-related valence toward the content. Eccles and Wigfield (2002) define interests as enduring affective orientations toward an object or class of objects resulting from the interaction between an individual and these objects. Objects of interests can be academic ideas, vocational or leisure time activities. Most of these objects carry a gender connotation or are part of gender stereotypes (e.g., Nosek et al., 2002; Ruble et al., 2006; Lagaert et al., 2017). The two areas of interest that this study will focus on are academic interests, i.e., in regard to school subjects, in particular German and mathematics, which have particularly gender-stereotyped connotations (Hannover and Kessels, 2004; Steffens and Jelenec, 2011; Makarova and Herzog, 2015), and vocational interests, i.e., in regard to occupational tasks and responsibilities. As a novel way to regard interests during early secondary school years, we integrate both academic and vocational interests into interest profiles. With the identification of profiles, we extent previous research. For example, by using this approach we can identify congruent and incongruent patterns of interests in multiple interest domains. German and mathematics as well as most vocations have gendered connotations (e.g., Garrett et al., 1977; Steffens and Jelenec, 2011). Accordingly, we expected gendered interest profiles and boys and girls – at the end of middle childhood – to differ in their interest profiles. Furthermore, we wanted to identify what predicts highly gender-stereotypical interests versus interests that are inconsistent with gender stereotypes. The student characteristics we focus on are children’s cognitive abilities, gender, and gender-role orientations. The goal of this study is to find interest profiles and investigate their connection to students’ individual characteristics in a representative large-scale assessment study. Thus, the research questions are explored with a sample of the German National Educational Panel Study (NEPS; Blossfeld et al., 2011).

## Theoretical Background

### Gender Differences in Academic and Vocational Interest

Academic and vocational interests have been found to predict educational and occupational attainments, aspirations, and decisions and to be even more important predictors than gender or grades (Lent et al., 1994, 2010; Volodina and Nagy, 2016).

For academic interests, Korhonen et al. (2016) found, for example, that interest in mathematics and reading was related to achievement in both genders. Further, the relationship between achievement and academic aspirations was mediated by interest in reading for girls and boys and by interest in mathematics for girls. Other studies of high school students found interest in mathematics to be a predictor of mathematics grades and chosen course level (Schiefele and Csikszentmihalyi, 1995; Köller et al., 2001).

According to Holland’s popular theory (1997), vocational interests can be categorized into six types or domains of interest. These six domains are realistic, investigative, artistic, social, enterprising, and conventional interests. Accordingly, this model is named the RIASEC model. Holland (1997) describes people holding realistic interests as preferring activities where they can manipulate objects, i.e., manual activities, and avoid academic activities. People with investigative interest prefer activities focusing on the “investigation of physical, biological, and cultural phenomena” (Holland, 1997, p. 22) as well as scientific and scholarly activities. Artistic interest is characterized by a preference for creation of different art forms and self-expression, while avoiding ordered activities. People holding social interests prefer activities that focus on other people rather than things. People with enterprising interest are characterized by their preference for activities from which they can attain economic gain, focusing on leadership and persuasive competencies. Lastly, people with conventional interests favor activities involving a clear structure while avoiding ambiguous tasks. These types of interest vary in strength in different people, while not being mutually exclusive to one another. Similar to academic interests, vocational interests have an impact on academic achievement (Warwas et al., 2009). Warwas et al. (2009) found in a sample of 11th grade students a relationship between students’ high realistic interest and higher mathematical literacy as well as a relationship between high artistic and social interests and lower mathematical literacy.

Stereotypes about school subjects being either feminine or masculine are still prevalent in children today. Mathematics is, for example, stereotyped as male whereas languages are stereotyped as female (Hannover and Kessels, 2004; Steffens and Jelenec, 2011; Makarova and Herzog, 2015). This gender stereotyping of children and adults has also been found for occupations (Garrett et al., 1977; White and White, 2006). Being an engineer has, for example, been found to be perceived as stereotypically male, while being an elementary school teacher is perceived as a stereotypically female occupation (White and White, 2006). This stereotyping of domains is mirrored in children’s interests. Regarding academic interest, several studies have shown that gender differences exist in reading and mathematics interests in the direction of girls having higher reading and lower mathematics interests and boys having higher mathematics and lower reading interests (Evans et al., 2002; Su et al., 2009; Chow and Salmela-Aro, 2011). Gender differences have also been found in empirical studies on the six types of vocational interest (Su et al., 2009; von Maurice and Bäumer, 2015). According to a meta-analysis by Su et al. (2009), men have generally higher realistic, investigative, and enterprising interests, whereas women have generally higher social, artistic, and conventional interests. The highest effect sizes for gender differences were found in realistic and social interests, the lowest effect sizes were found in enterprising interest, although findings have been inconsistent (Proyer and Häusler, 2007). Thus we have concluded from this empirical evidence that gender-stereotyped interests should also be present in our study.

### Gender-Stereotypical Interest Profiles

In this study, we aim at extending previous findings on gender differences in various interest domains by focusing on gender-stereotypical interest profiles. We assume that the genders not only differ in the strength of their interest in various subject domains but that we can identify clusters of interests, with the probability of belonging to one of the clusters varying systematically according to the student’s gender. Such a person-orientated investigation of profiles adds to existing variable-centered research because the approach allows to identify gender congruent and gender incongruent patterns, as well as patterns indicating strong or weak interest across subject domains, irrespective of the subjects’ gender connotation, thus disentangling the impact of gender stereotypes and of overall strength of interest.

We expect to find interest profiles in sixth grade students of combined academic and vocational interests. These interest profiles should either be differentiated by the level of interests, i.e., high and low interests, or by gender-stereotypical content, i.e., male- or female-stereotypical interests. To this end, instead of just comparing average scale scores, students will be clustered into latent interest profiles which show the relative level of each interest domain to each other. Profiles of interest should be investigated during students’ compulsory school years, since during high school, a broad range of skills in different subjects needs to be acquired, compared to a later specialization of skills during university studies or at work.

Interest profiles with gendered connotations have been found in both academic and vocational interests, meaning differentiated interests in male and female interest domains. In a sample of students in 11th grade, Warwas et al. (2009) examined vocational interest profiles and compared them to students’ self-reports on single interest scales. Both methods produced comparable results in regard to predicting students’ mathematical literacy. However, once covariates were included in the model, only interest profiles still added to the prediction of mathematical literacy. The authors concluded that interest profiles were a more reliable predictor of mathematical literacy than just interest scale scores. Warwas et al. (2009) did not, however, explore interest profiles in relation to students’ gender. Chow and Salmela-Aro (2011) found in a study with a sample of ninth grade students gender-specific task-value profiles of a “high-math-and-science” profile which was dominated by boys, and a “low-math-and-science” profile which was dominated by girls. Additionally, Viljaranta et al. (2009) reported in a sample of predominantly 16-year-old students six profiles of task values differentiated by overall level and gender-stereotypical content, including a high profile, a low profile, and gender-stereotypical profiles. Girls were overrepresented in the “multi-motivated” profile and “practical skills and language-motivated” profile, whereas boys where overrepresented in the “low-motivated,” “math and science-motivated,” and “practical skills-motivated” profiles. These profiles predicted students’ academic and vocational attainment expectations.

Interest profiles found in previous studies (Viljaranta et al., 2009; Warwas et al., 2009; Chow and Salmela-Aro, 2011) show gender-stereotypical patterns, i.e., profiles in which interests are high in both mathematics and sciences and low in languages and, *vice versa*, profiles with low interest in mathematics or high interest in languages. Most studies investigating interest profiles have focused on older adolescents or adults, probably because they are already actively involved in making vocational decisions. There has been little research thus far on vocational or pre-vocational interest profiles in children. Furthermore, previous research in children has focused rather on interest scores than profiles. According to the circumscription and compromise theory by Gottfredson (e.g., Gottfredson and Lapan, 1997; Gottfredson, 2002), children start valuing the sextype or gender stereotype of occupations in elementary school and eliminate occupations as options accordingly. Focusing on the development of vocational interests in younger age groups, von Maurice and Bäumer (2015) found that gender differences in vocational interest domains are already present in 9-year-old children or third graders and become even stronger between grades three and five. We therefore expected gendered vocational tasks would also be reflected in young adolescent students’ interest profiles.

In summary, in this study, two interest areas, academic and vocational, will be investigated. Both have been found to predict important educational and vocational outcomes, such as achievement and choices (Warwas et al., 2009; Korhonen et al., 2016). Empirical studies about these interests have also found gender difference in the respective sub-domains (Su et al., 2009; Chow and Salmela-Aro, 2011). Interest profiles have been shown to be a valid alternative to absolute scale scores in predicting vocational and academic outcomes (Warwas et al., 2009) and interest profiles of adolescent students have been found to be generally either gender-stereotyped or high or low in all interests (Viljaranta et al., 2009; Chow and Salmela-Aro, 2011). In the current study, we want to investigate whether gendered interest profiles can already be found in approximately 12-year-old sixth grade children. We expected to find high, low as well as gender-stereotypical interest profiles.

### The Impact of Students’ Gender-Role Orientation and Gender on Gender-Stereotypical Interest Profiles

As gender-specific interest profiles are based on gender stereotypes, we expected that students’ interest profiles would be interrelated with their attitudes toward gender roles, i.e., their gender-role orientation. An individual’s gender-role orientation describes internalized societal norms and expectations regarding traditional gendered behaviors. Labor therefore is divided between the genders – men are traditionally characterized as the breadwinners while women are designated to take care of the household and children – and people also perceive other behaviors as more or less appropriate or desirable depending on the actor’s biological sex. People holding a *traditional gender-role orientation* differentiate between the appropriateness of traits, attitudes, and behaviors for either men or women and, therefore, also endorse gendered division and distribution of labor (e.g., Athenstaedt, 2000). People holding an *egalitarian gender-role orientation*, however, advocate for equal occupational and behavioral opportunities and rights for both genders and reject differences between the genders as described by gender stereotypes (e.g., Athenstaedt, 2000).

Students’ gender-role orientation should be related to their level of interest, i.e., low interest or high interest profiles. In particular, we expected that students with traditional attitudes toward gender roles would more likely be represented in low interest profiles because they can be expected to be less inclined to strive for academic success. In a study by Ehrtmann and Wolter (2018), girls were restricted in their competence development between grades five and seven in the domains of reading and mathematics only when they showed traditional attitudes. Hadjar et al. (2012), however, found a relationship between traditional gender role attitudes and lower grades for boys and girls in eighth grade. We assume these findings are due to lower academic aspirations of students with a traditional gender-role orientation.

A traditional gender-role orientation has been found to be related to academic achievement and vocational interests (Tokar and Jome, 1998; Steffens and Jelenec, 2011; Hadjar et al., 2012; Plante et al., 2013). In particular, Tokar and Jome (1998) found that men who endorse male gender roles report more traditional vocational interests and career choices. Ehrtmann and Wolter (2018) showed that especially traditional girls are restricted in their competence development in mathematics and reading between grades five and seven compared to boys and egalitarian girls. Egalitarian girls therefore had an advantage over traditional girls in their competence development in both mathematics and reading, while egalitarian boys had an advantage over traditional boys only in reading. Traditional girls therefore displayed lower competences than egalitarian girls in a traditional male domain, mathematics, as well as a traditional female domain, reading; boys were however only affected by a traditional orientation in the traditional female domain, reading. Concerning occupational aspirations, women with traditional attitudes toward gender roles also show more traditional aspirations; the same relationship could, however, not be found for men, who were therefore not more likely to have aspirations to work in occupations with more women even when they showed egalitarian attitudes (Baird, 2012). For our study, we assume that girls with an egalitarian gender-role orientation would therefore more likely show male-stereotypical interests than girls with a traditional orientation, whereas boys would not have a higher probability of showing a counter-stereotypical (i.e., female-stereotyped) interest profile with neither traditional nor egalitarian gender-role orientation.

Since a traditional gender-role orientation was associated with lower achievement (e.g., Hadjar et al., 2012; Ehrtmann and Wolter, 2018), we expected that children’s gender-role orientations should also be related to overall interest levels. Traditional gender roles for girls are less consistent with a successful academic or professional career than for boys, in particular with respect to a career in male-connoted subjects or domains. We therefore expected girls to profit particularly strongly from an egalitarian orientation and that a traditional gender-role orientation should strengthen girls’ (but not boys’) relative preference for gender-stereotypical domains over counter-stereotypical ones (e.g., Baird, 2012; Ehrtmann and Wolter, 2018).

In addition to children’s gender-role orientations, their gender should play a role in their stereotypical interests. We expected gender to be a main factor explaining students’ interest profiles (e.g., Viljaranta et al., 2009).

It is especially notable that children and adults experience sanctions or stigmatization when they do not act in accordance with traditional gender roles or stereotypes (Blakemore, 2003; Heilman et al., 2004; Rudman and Mescher, 2013). According to Social Role Theory (e.g., Eagly et al., 2000), gender stereotypes develop from the division of labor between men and women. Men more often act in high-status positions and are therefore associated with agency while women more often act in nurturant roles and are therefore associated with communion. According to social-cognitive theory (Bussey and Bandura, 1999), children acquire these gender stereotypes and gender roles through social modeling, enactive experience, and direct instruction. Especially modeling is a strong mechanism through peers, parents, or media (Eccles et al., 1990; Witt, 1997, 2000; Collins, 2011). Accordingly, children also show greater interests in occupations that are associated with their own gender (Liben et al., 2001; Weisgram et al., 2010). This is also evident in an ongoing gender-segregated labor market (e.g., Hausmann and Kleinert, 2014).

Reasons for this gender segregation of interests and occupations might lie in the social sanctioning of counter-stereotypical behavior. Studies have shown that even though both boys and girls suffer from sanctions, the mechanisms behind these sanctions may differ. According to Blakemore (2003), boys are socially sanctioned for looking feminine and for feminine activities, girls, however, are only sanctioned for masculine activities. Even though both genders are sanctioned for counter-stereotypical activities, boys have the additional disadvantage of also being sanctioned for their looks. In a study of adults, Heilman and Wallen (2010) found that men in gender-atypical jobs are perceived as more ineffectual and are less respected, whereas women in gender-atypical jobs are more disliked and derogated. Likewise, men who endorse gender-egalitarian attitudes are also seen as feminine, weak, and likely to be gay (Rudman et al., 2012), which is due to their association with a low-status group, i.e., women. Moreover, it seems that men are underrepresented in female-dominated occupations compared to women in male-dominated occupations (Croft et al., 2015). Croft et al. (2015) argue that this underrepresentation is a consequence of the perceived lower status of communal roles and female occupations. Children already perceive male-connoted occupations as higher in status than female occupations (Liben et al., 2001). In addition to women’s occupations being perceived as lower in status, children find it more difficult to process information about men in counter-stereotypical occupations than about women in counter-stereotypical occupations (Wilbourn and Kee, 2010). Following this argumentation, boys should be less prominently represented in the female-stereotypical interest profile than girls in the male-stereotypical interest profile due to sanctions against their counter-stereotypical behavior.

### The Interrelatedness of Cognitive Skills and Interests

Lastly, we assume that students’ cognitive skills play a role in their level of interests. Low reasoning ability should be connected to a low interest profile while high reasoning ability should be connected to a high interest profile.

The relationship between cognitive skills and interests can be explained by the social cognitive theory of career development (Lent et al., 1994), which presumes motivational factors, like self-efficacy or expectations of outcomes, play a role in the development of academic and vocational interests together with social factors. Interests then have a relationship with performance attainments, which in turn affect interests, making the relationship between interests and performance attainments reciprocal.

Following this line of reasoning, different studies have found links between domain-specific achievement and interest. Achievement is certainly not just a cognitive factor but heavily influenced by cognitive abilities (e.g., Rohde and Thompson, 2007). Korhonen et al. (2016) found that achievement in mathematics and reading was related to interest in the according domain. Another study (Marsh et al., 2005) reported small reciprocal links between students’ mathematics interest and achievement as well as self-concept. Denissen et al. (2007) showed that students felt more competent and interested in domains in which they also showed high achievement and felt their personal strength.

Päßler et al. (2015) investigated the relationship between cognitive skills and vocational interests in students and adults in a meta-analysis. The results showed that a general factor of intelligence correlated positively, in both boys and girls, with realistic, investigative, and conventional interests, negatively with artistic and social interests, and not at all with enterprising interest. Furthermore, in a study by Schoon and Polek (2011), general cognitive ability was found to be a predictor of general higher occupational aspirations.

Overall, there seems to be a connection between cognitive factors, such as achievement and cognitive abilities, and interests, even though the effects are not consistent. Since our aim is to investigate characteristics of students in certain combined interest profiles rather than single, domain-specific interests, we expect cognitive abilities to be related to students’ interest profiles in the direction of high cognitive abilities being associated with a high interest profile.

## Research Aims and Hypotheses

We expected to find gender-stereotypical interest profiles in girls and boys in middle childhood. These interest profiles should incorporate both academic and vocational domains and reflect gender-stereotypical patterns. We further expected that students’ gender and gender-role orientation should be related to their interest profiles. Boys should be less likely to display female-stereotypical interest profiles than girls display male-stereotypical interest profiles since boys should expect greater sanctions than girls when interested in non-gender-stereotypical domains. Gender-role orientation of students should be related to whether students express low interests in all domains or not. It should further be of relevance for girls who should only express interests in male-stereotyped domains when they also express egalitarian gender role views.

### Interest Profiles

We expect to find four interest profiles of combined academic and vocational interests. A “low interest profile” is characterized by only weak interest in all domains. A “high interest profile” is characterized by strong interests in all domains.

Furthermore, we expected to find two gender-stereotypical interest profiles. The “female-stereotypical interest profile” should be characterized by higher interest in female-stereotypical interest domains than in male-stereotypical interest domains. Students in this profile are expected to show high interest in German, artistic, social, and conventional domains, and low interest in mathematical, realistic, investigative, and enterprising domains. The “male-stereotypical interest profile” should be characterized by higher interest in male-stereotypical interest domains than in female-stereotypical interest domains. Students in this profile are expected to show higher interest in mathematical, realistic, investigative, and enterprising domains and low interest in German, artistic, social, and conventional domains.

### Profile-Specific Hypotheses

Cognitive skills in the form of reasoning ability, students’ gender, and students’ gender-role orientation are expected to be associated to the probability of students belonging to a certain interest profile compared to other interest profiles as specified below:

#### Low Interest Profile

Students, regardless of gender, with more traditional gender-role orientations (H1a) or low reasoning abilities (H1b) have a higher probability of belonging to the low interest profile compared to other interest profiles.

#### High Interest Profile

Students, regardless of gender, with more egalitarian gender-role orientations (H2a) or high reasoning abilities (H2b) have a higher probability of belonging to the high interest profile compared to other interest profiles.

#### Female-Stereotypical Interest Profile

Girls have a higher probability than boys of belonging to the female-stereotypical interest profiles (H3a); boys are rarely represented in this profile since they would face greater obstacles than girls to express gender-atypical interests (H3b).

Regardless of gender, students with high reasoning abilities are more likely to belong to the female-stereotypical interest profile than to the low interest profile (H3c).

#### Male-Stereotypical Interest Profile

Boys have a higher probability than girls to belong to the male-stereotypical interest profile (H4a). For girls, the probability of belonging to this profile depends on their gender-role orientation (H4b). Girls are more likely to belong to this profile than to other profiles only when they are also egalitarian. Girls are therefore represented in the male-stereotypical interest profile in higher proportions than boys in the female-stereotypical interest profile. Boys, however, are more likely to belong to this profile than to other profiles regardless of their gender-role orientation.

Regardless of gender, students with high reasoning abilities are more likely to belong to the male-stereotypical interest profile than to the low interest profile (H4c).

## Materials and Methods

### Data

This study was conducted with data from the German National Educational Panel Study (NEPS). The NEPS is a longitudinal panel study on educational trajectories in Germany following a multi-cohort sequence design (Blossfeld et al., 2011). In particular, this study used data from the NEPS starting cohort three, which started assessments with fifth grade students in 2010.

For registered researchers, the NEPS provides Scientific Use Files, which include data on competence tests of students and student, parent and teacher questionnaires as well as cohort information. The study was conducted as an educational survey. A large number of other measures was administered during the whole survey, ranging from socio-demographics to motivational measures. More information and all scales can also be found on the NEPS website: https://www.neps-data.de/en-us/home.aspx. Written informed consent was given by the students and their parents. Participants were told that they could stop the survey at any time without any disadvantage.

### Sample

The sample consisted of 4,457 students (49.2% female), with a mean age of *M* = 11.88 years (SD = 0.50) in grade six; 24.7% of the students had a migration background (i.e., either they or a parent were born abroad). Germany applies achievement tracking in their secondary education system. Depending on the state, after 4 or 6 years of primary school, students are placed into different school types which, after graduation, either provide the option of pursuing vocational training or of attending higher education at the university level. Our sample consists of an oversampling of students in the highest educational track (“Gymnasium”) with 46% of students belonging to this track versus 34% of students according to the German Federal Statistical Office (2012).

### Research Instruments

Measurement points of academic interests, vocational interests, and gender-role orientations were all located in sixth grade, whereas only students’ reasoning ability was assessed in grade five. Reasoning ability is assumed to be a fairly stable construct (Strand, 2004); for this reason, reasoning ability being measured a year prior to the other constructs should not pose a problem. All measures were developed by an interdisciplinary team of item developers working for the NEPS and were extensively pretested (Blossfeld et al., 2011).

#### Academic Interest in German

Academic interest in the school subject German, which includes German language and literature, was measured by four items (e.g., “I really enjoy learning more about myself and the world through reading books.”), with answers ranging from *does not apply at all* to *applies completely* on a 4-point Likert scale, ranging from 1 to 4. The scale showed good reliability, *α* = 0.74, and the mean for the sample was *M* = 2.21 (SD = 0.77). This scale was adapted from Baumert et al. (2003).

#### Academic Interest in Mathematics

Academic interest in mathematics was measured with four items (e.g., “I enjoy puzzling over a mathematical problem.”), with answers ranging from *does not apply at all* to *applies completely* on a 4-point Likert scale, ranging from one to four. The scale showed good reliability, *α* = 0.76, and the mean for the sample was *M* = 2.32 (SD = 0.71). This scale was adapted from Baumert et al. (2003).

#### Vocational Interests

Vocational interests were measured by the Interest Inventory Life Span (IILS-I), a scale developed for the NEPS, assessing all six of Holland’s interest domains (Wohlkinger et al., 2011). Each domain was assessed by three items with the question “How much are you interested in the following things?” and answers ranged from *I have little interest in that; I do not like doing that* to *I am very interested in that; I like doing that* on a 5-point Likert scale, ranging from 1 to 5. Realistic interest (“building or assembling things,” “watching someone repair an electrical device,” and “working with metal or wood/creating things from metal or wood”) showed a reliability of *α* = 0.68, and a mean of *M* = 2.99 (SD = 1.08). Investigative interest (“watching a science show,” “conducting experiments in a test laboratory,” and “viewing things through a microscope”) showed a reliability of *α* = 0.64, and a mean of *M* = 3.10 (SD = 1.01). Artistic interest (“drawing pictures,” “designing something artistically,” and “playing with clay or play dough”) showed a reliability of *α* = 0.65, and a mean of *M* = 3.14 (SD = 1.03). Social interest (“helping others feel comfortable,” “help sick people,” and “caring for children or adults in need”) showed a reliability of *α* = 0.76, and a mean of *M* = 3.40 (SD = 0.91). Enterprising interest (“negotiating with other people,” “being a leader of a group,” and “telling other people what they should do”) showed a reliability of *α* = 0.62, and a mean of *M* = 2.64 (SD = 0.96). Conventional interest (“keeping lists or records of things,” “counting and sorting things,” and “tidying up a closet”) showed a reliability of *α* = 0.53, and a mean of *M* = 2.34 (SD = 0.87)^2^.

#### Gender-Role Orientation

Students’ gender-role orientation was measured by four items (“Boys and girls should have the same chores at home.”; “Girls can handle technical devices just as well as boys.”; “Girls should be able to learn the same professions as boys.”; “Men are better suited for some professions than women.”) Answers for this scale ranged from *completely agree* to *completely disagree* on a 4-point Likert scale, ranging from 1 to 4. Higher scores on the scale signify more egalitarian gender-role orientations, whereas lower scores signify more traditional gender-role orientations. The scale had good reliability, *α* = 0.72, and a mean of *M* = 2.79 (SD = 0.78).

#### Reasoning Ability

Reasoning as one aspect of basic cognitive skills was assessed in grade five using a matrices test (NEPS-MAT) which was developed for the NEPS (Haberkorn and Pohl, 2013; Brunner et al., 2014). The test consisted of 12 items which are described by Haberkorn and Pohl (2013, p. 2) as “horizontally and vertically arranged fields in which different geometrical elements are shown – with only one field remaining free. The logical rules on which the pattern of the geometrical elements is based have to be deduced in order to be able to select the right complement for the free field from the offered solutions.” The mean of the provided sum scores for this sample was *M* = 7 (SD = 2.61).

### Analysis

In order to test the presented hypotheses, a latent profile analysis was conducted using Mplus Version 8 (Muthén and Muthén, 2017). Mathematical, German, and vocational interests were standardized because they were measured on different scales. Latent profile solutions from one to six profiles were compared. The aim of our study was to find clusters that are described by combinations of interests with regard to multiple academic (German, mathematics) and vocational (RIASEC model) interest domains. To our knowledge, a latent profile analysis is an appropriate way to include the joint interests of a person by allocating latent interest profiles and calculating an individual’s probability of membership or using the most likely group as outcome. The decision for using latent profiles is therefore based on a joint consideration of academic and vocational interests for an individual. We still use continuous information of each variable which is then used to allocate the latent profiles. The advantage of this approach is that we did not need to aggregate the academic and vocational interest in a joint variable, but rather look at them from a more holistic perspective to consider the probable profile membership of each person.

To test the profile-specific hypotheses, the automatic three-step method implemented by Mplus through the R3STEP command was used (Asparouhov and Muthén, 2014). In this procedure, three steps were conducted. In a first step, we computed a latent profile analysis using the latent profile indicators, in this case the interest domains. In a second step, the most likely profile membership was established for each observation, in this case for each student, using the latent class posterior distribution obtained during the first step. In the third step, auxiliary variables (i.e., the predictor variables) were included; the profile memberships were fixed according to the previous step and used in a multinomial logistic regression as dependent variables. As independent variables, students’ reasoning ability, gender, gender-role orientation, and the interaction of students’ gender and gender-role orientation were included in the model^3^. Germany has a highly tracked secondary school system. However, we decided not to include school tracks as a control variable since tracking should be closely related to reasoning ability. Furthermore, school tracks are assumed to be related to mean level differences in interest domains, but are not expected to be relevant for the clustering of interest profiles. Reasoning ability and gender-role orientation were grand-mean centered beforehand. The Mplus syntax and model output for the four profile solutions of the latent profile analysis are available under: https://osf.io/wv2at/?view_only=9dcfea10b3144c0fb49f359f7aac17d9

Cases with missing values on either dependent or independent variables were excluded from the analyses to ensure that the sample was identical for the latent profile analysis and the following multinomial logistic regression analyses.

## Results

### Descriptive Results

Means and standard deviations for all variables used in the latent profile analysis, separated by gender, as well as effect sizes (Cohen’s *d*) of gender differences are displayed in Table 1. According to independent sample *t*-tests, gender differences (gender was effect-coded in all analyses: boys = −0.5, girls = 0.5) were significant in all interest domains, in students’ gender-role orientations, and in their reasoning abilities.

**Table 1:** Means and standard deviations for interest domains, i.e., the latent class indicators, and predictor variables for boys and girls separately.

	Overall *M* (SD)	Boys *M* (SD)	Girls *M* (SD)	Effect size *d*
Realistic	2.99 (1.08)	3.42 (1.02)	2.54 (0.95)	0.89*
Investigative	3.10 (1.01)	3.24 (1.03)	2.95 (0.97)	0.29*
Artistic	3.14 (1.03)	2.73 (0.98)	3.56 (0.90)	−0.89*
Social	3.40 (0.91)	3.15 (0.90)	3.67 (0.84)	−0.60*
Enterprising	2.84 (0.96)	3.08 (0.96)	2.59 (0.89)	0.52*
Conventional	2.34 (0.87)	2.22 (0.83)	2.47 (0.89)	−0.29*
German	2.32 (0.71)	2.21 (0.70)	2.43 (0.71)	−0.32*
Mathematics	2.21 (0.77)	2.32 (0.78)	2.09 (0.75)	0.30*
Gender-role orientation	2.79 (0.78)	2.44 (0.76)	3.15 (0.62)	−1.04*
Reasoning ability	7.00 (2.61)	7.10 (2.65)	6.90 (2.57)	0.07*

Effect sizes for gender differences.

* p < 0.05.

Higher scores for gender-role orientation = more egalitarian attitudes.

Boys’ interest was higher in realistic [*t*(4403) = 29.66, *d =* 0.89], investigative [*t*(4410) = 9.51, *d =* 0.29], enterprising [*t*(4332) = 17.13, *d =* 0.52], and mathematics [*t*(4386) = 9.95, *d =* 0.30] interest domains. Girls had higher interests in artistic [*t*(4346) = −29.25, *d =* −0.89], social [*t*(4346) = −19.85, *d =* −0.60], conventional [*t*(4327) = −9.51, *d =* −0.29], and German [*t*(4350) = −10.66, *d =* −0.32] domains. Regarding predictor variables, girls had higher scores in gender-role orientation [*t*(4341) = −34.27, *d =* −1.04], i.e., a more egalitarian orientation than boys. Boys had higher cognitive basic skills (i.e., reasoning) than girls [*t*(4455) = 2.50, *d =* 0.07].

The bivariate correlations between all latent class indicators in the subsequent latent class analysis, i.e., all interest domains, and the predictor variables gender-role orientation, and reasoning ability can be found in Table 2. Notably, an egalitarian gender-role orientation correlated positively with the female-connoted interest domains of artistic, social, conventional, and German interests, whereas it correlated negatively or not all with the male-connoted interest domains of realistic, investigative, enterprising, and mathematics interests. Reasoning ability was positively but weakly correlated with realistic, investigative, artistic, and mathematics interests.

**Table 2:** Correlations between all latent class indicators and predictor variables.

	1	2	3	4	5	6	7	8	9
Realistic	-								
Investigative	0.51*	-							
Artistic	0.13*	0.27*	-						
Social	0.09*	0.25*	0.34*	-					
Enterprising	0.24*	0.13*	0.02	0.02	-				
Conventional	0.21*	0.25*	0.33*	0.32*	0.15*	-			
German	0.34*	0.35*	0.12*	0.18*	0.10*	0.35*	-		
Mathematics	0.15*	0.34*	0.36*	0.40*	0.03	0.39*	0.31*	-	
Gender-role orientation	−0.12*	0.07*	0.29*	0.29*	−0.16*	0.13*	−0.00	0.21*	-
Reasoning ability	0.07*	0.11*	0.03*	−0.00	0.01	−0.02	0.06*	0.01	0.11*

* p < 0.05.

### Results of Latent Profile Analysis

**Figure 1:**
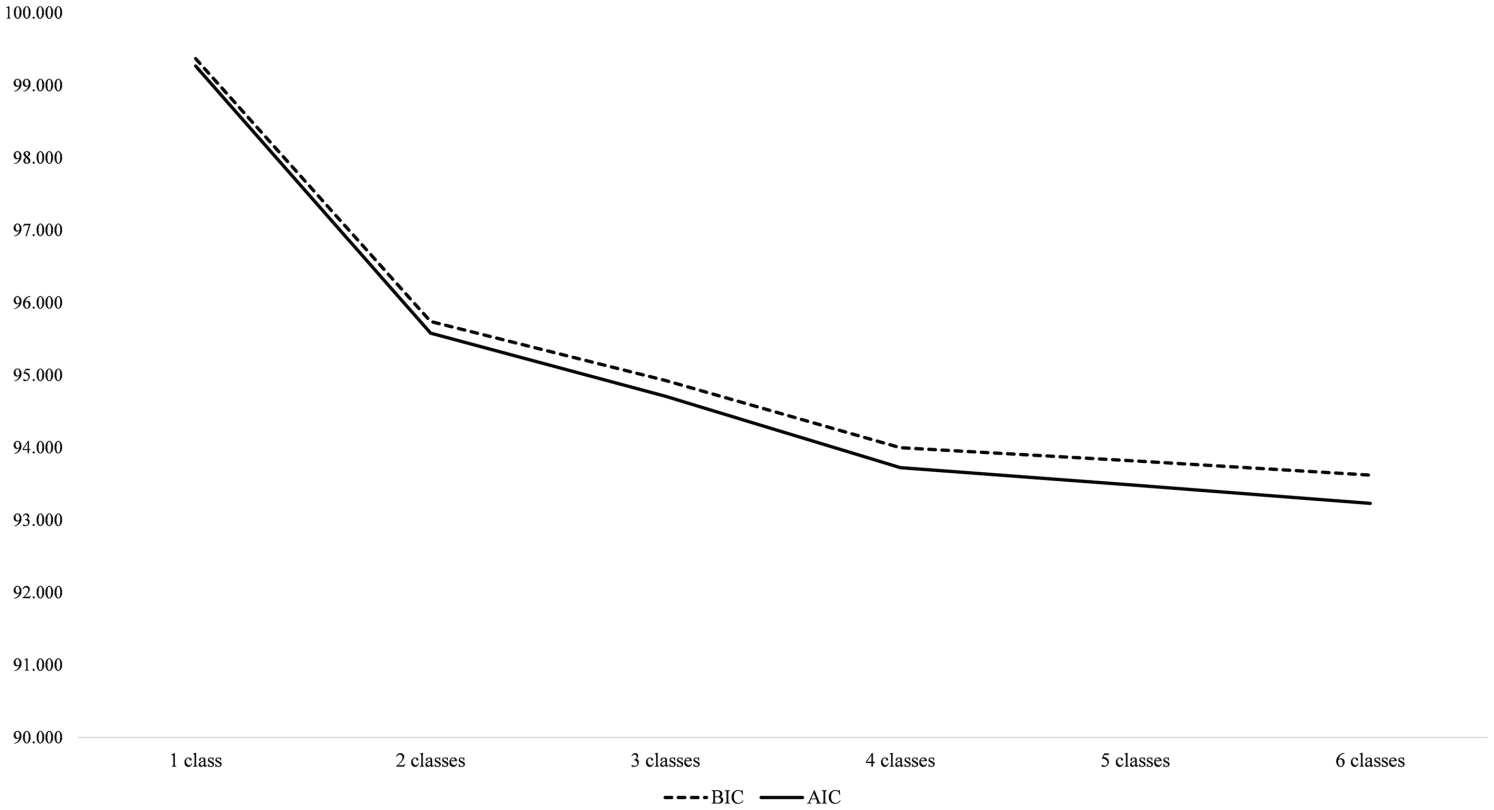
AIC and BIC values of one- to six-class solutions.

Before using the previously described three-step method to test the profile-specific hypotheses, simple latent profile analyses with profiles differing from one to six were conducted to determine if the presumed four class solution was acceptable. The Vuong-Lo-Mendell-Rubin likelihood ratio test (Vuong, 1989; Lo et al., 2001) showed that a four-profile model had a better fit compared to a three-profile model (adjusted value = 990.95, *p* < 0.01), the fit of the four-profile model was however worse compared to a five-profile model (adjusted value = 237.15, *p* = 0.03). Figure 1 shows the Bayesian information criterion (BIC) and the Akaike’s information criterion (AIC) of each latent profile analysis. The BIC and the AIC were used to compare the different models (e.g., Nylund et al., 2007). As can be seen, there were two bends in the slopes, one at a two-profile solution and one at a four-profile solution. Since the AIC and the BIC did not decrease considerably after the four-profile solution (four profiles: BIC = 93,999; AIC = 93,724; five profiles: BIC = 93,816; AIC = 93,483), and the profile solutions with a larger number of profiles did not contribute any more theoretical value, but merely level differences in the profiles, the four-profile solution was used in further analyses^4^. Through this procedure, each student was given a classification probability for each profile.

**Figure 2:**
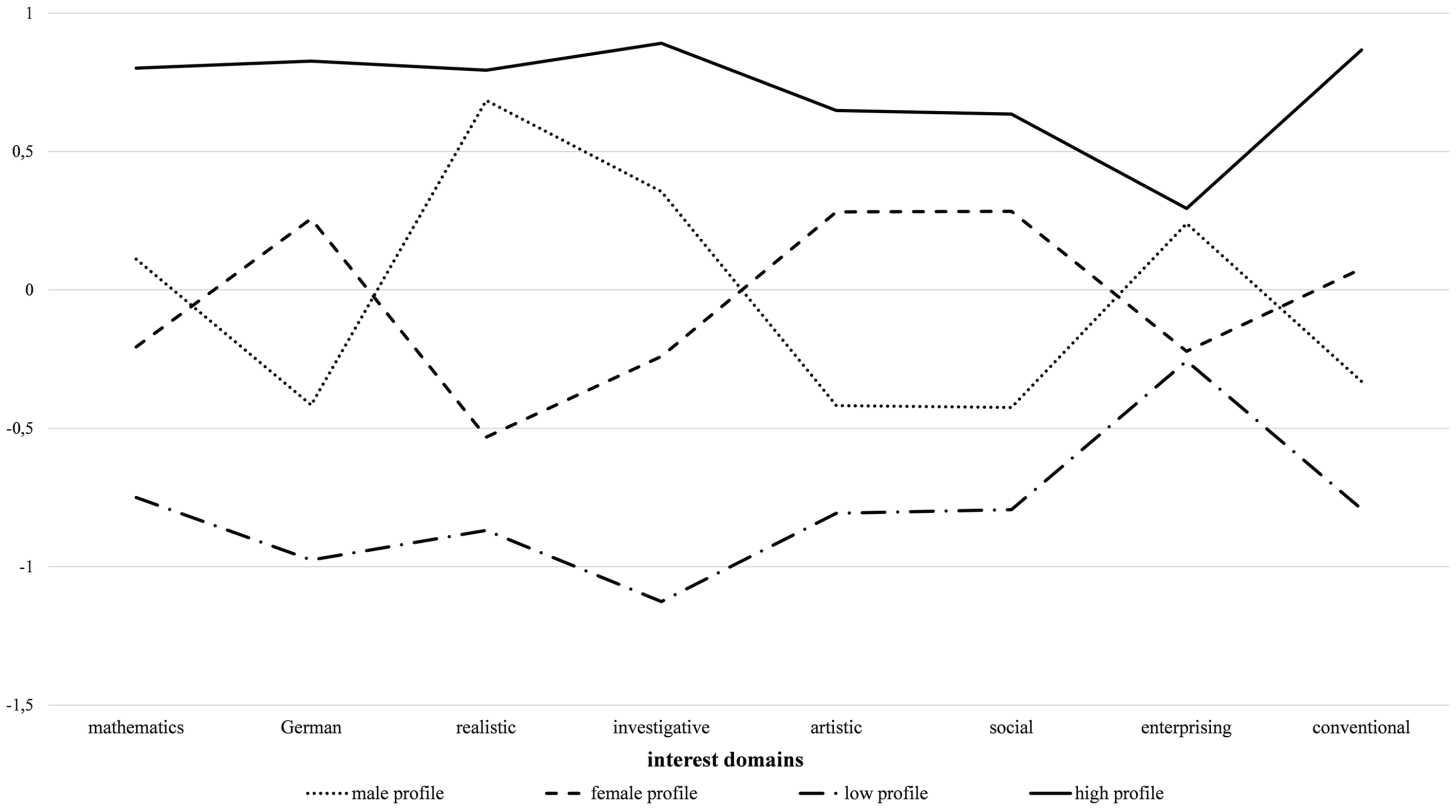
Graphical display of the results of the four-profile latent profile model.

Figure 2 displays the results of the four-profile latent profile model. For the analyses, all interest variables were z-standardized to account for the difference in scales. As was proposed in the hypotheses, two undifferentiated interest profiles and two gender-stereotypical interest profiles were confirmed. The undifferentiated interest profiles included one profile with high interests (“high interest profile”) in all domains and one profile with low interests in all domains (“low interest profile”). The gender-stereotypical interest profiles included one “male-stereotypical interest profile” with relatively high interests in the mathematics, realistic, investigative, and enterprising domains. This profile is further characterized by relatively low interests in the German, artistic, social, and conventional domains.

The second gender-stereotypical interest profile can be described as the “female-stereotypical interest profile.” This profile was identified by relatively high interests in the German, artistic, social, and conventional domains and relatively low interest in the mathematics, realistic, investigative, and enterprising domains.

Table 3 shows the distribution of the four profiles for the whole sample as well as for boys and girls. The low interest profile covered the smallest proportion of 17.2% of all students, the female-stereotypical interest profile was the largest profile with a proportion of 36.8% of all students. For boys, the largest profile was the male-stereotypical interest profile, with a proportion of 41.7% of all boys belonging to it. For girls, the female-stereotypical interest profile was the largest profile, with a proportion of 57.2% of all girls belonging to it. Interestingly, still 17% (386 cases) of the boys belonged to the female-stereotypical interest profile, whereas only 6.4% (141 cases) of the girls belonged to the male-stereotypical interest profile. This difference between boys and girls being categorized in the profile opposite of gender stereotypes was significant, *t*(3909) = 11.20, *d* = 0.36. Boys were therefore significantly more often categorized into the female-stereotypical interest profiles than girls into the male-stereotypical interest profile. This finding goes against our hypothesis **H3b**, which states that boys should rarely be presented in the female-stereotypical profile.

**Table 3:** Frequencies for the four interest profiles for the whole sample and separate for boys and girls.

	Overall (%)	Boys (%)	Girls (%)
Low profile	17.2	18.2	16.1
High profile	21.7	23.1	20.2
Female profile	36.8	17.0	57.2
Male profile	24.3	41.7	6.4

Independent sample *t*-tests showed significant gender differences in the classification probabilities between boys and girls in the high [*t*(4455) = 2.36, *d =* 0.07], female [*t*(3927) = −35.08, *d =* −1.12], and male [*t*(3427) = 34.65, *d =* 1.18] interest profiles. Girls had a higher probability than boys to be categorized in the female interest profile, boys had a higher probability than girls to be categorized in the male and high interest profiles. The gender difference in the classification probability in the low interest profile was not significant [*t*(4448) = 1.94].

### Results of Logistic Regressions

In the next step, the three-step method described above was implemented to test the profile-specific hypotheses. The results of the derived logistic regressions are shown in Table 4. All significant results are reported, but only the results relevant to the hypotheses were examined in more detail.

**Table 4:** Results of multinomial logistic regression analyses.

Ref. class			*b*	SE	OR	CI
**High**						
	Low	Intercept	−0.315	0.071*	0.73	0.63/0.84
		Reasoning	−0.053	0.022*	0.95	0.90/0.99
		Gender	0.519	0.142*	1.68	1.27/2.22
		GRO	−0.839	0.097*	0.43	0.36/0.52
		IA	0.292	0.192	1.34	0.92/1.95
	Female	Intercept	0.127	0.087	1.14	0.96/1.35
		Reasoning	0.013	0.023	1.01	0.97/1.06
		Gender	2.298	0.175*	9.95	7.06/14.03
		GRO	−0.076	0.115	0.93	0.74/1.16
		IA	−0.519	0.231*	0.60	0.38/0.94
	Male	Intercept	−2.775	1.822	0.06	0.00/2.22
		Reasoning	0.100	0.025*	1.11	1.05/1.16
		Gender	−6.631	3.648	0.00	0.00/1.68
		GRO	−1.821	0.868*	0.16	0.03/0.89
		IA	−2.364	1.737	0.09	0.00/2.83
**Low**						
	Female	Intercept	0.441	0.091*	1.55	1.30/1.86
		Reasoning	0.067	0.024*	1.07	1.02/1.12
		Gender	1.779	0.184*	5.92	4.13/8.50
		GRO	0.762	0.119*	2.14	1.70/2.71
		IA	−0.810	0.238*	0.44	0.28/0.71
	Male	Intercept	−2.460	1.820	0.09	0.00/3.03
		Reasoning	0.153	0.025*	1.17	1.11/1.22
		Gender	−7.150	3.641	0.00	0.00/0.99
		GRO	−0.982	0.869	0.37	0.07/2.06
		IA	−2.656	1.739	0.07	0.00/2.12
**Female**						
	Male	Intercept	−2.902	1.820	0.05	0.00/1.94
		Reasoning	0.086	0.030*	1.09	1.03/1.16
		Gender	−8.929	3.648*	0.00	0.00/0.17
		GRO	−1.744	0.868*	0.17	0.03/0.96
		IA	−1.845	1.738	0.16	0.01/4.77

* p < 0.05.

GRO, gender-role orientation; IA, interaction between gender and GRO.

### Hypotheses H1a and H1b

The probability of being categorized into the low interest profile over the high interest profile (*b* = −0.84, SE = 0.10) or the female-stereotypical interest profile (*b* = 0.76, SE = 0.12) was higher with an increasing traditional gender-role orientation. The probability of being categorized into the low interest profile over the male-stereotypical interest profile, however, did not depend on students’ gender-role orientation (*b* = −0.98, SE = 0.87). Hypothesis **H1a**, that students with a traditional orientation are more likely represented in the low interest profile compared to other profiles, could therefore not be confirmed.

As for **H1b**, the probability of being categorized into the low interest profile over all other profiles was higher with a decreasing reasoning ability, confirming the hypothesis (compared to high: *b* = −0.05, SE = 0.02; female: *b* = 0.07, SE = 0.02; male: *b* = 0.15, SE = 0.03).

### Hypotheses H2a and H2b

There was a higher probability of being categorized into the high interest profile than into the low (*b* = 0.84, SE = 0.10) and male-stereotypical interest profiles (*b* = 1.82, SE = 0.87) with an increasingly egalitarian gender-role orientation. However, there was no equivalent result for the probability compared to the female-stereotypical interest profile (*b* = 0.08, SE = 0.12). Hypothesis **H2a**, that students with an egalitarian orientation are more likely represented in the high interest profile compared to other profiles, was therefore not confirmed.

For reasoning ability (**H2b**), the pattern was different: there was a higher probability of being categorized into the high interest profile than into the low interest profile (*b* = 0–05, SE = 0.02) with higher reasoning ability. The probability of belonging to the high interest profile compared to the male-stereotypical interest profile (*b* = −0.1, SE = 0.03), however, decreased with higher reasoning abilities. There was no effect of reasoning abilities on the probability of belonging to the high interest profile compared to the female-stereotypical interest profile (*b* = −0.01, SE = 0.02). The hypothesis (H2b), that students with a high reasoning ability more likely belong to the high interest profiles compared to other profiles, could not be confirmed.

### Hypotheses H3a, H3b, and H3c

Hypothesis **H3a**, that girls most likely belong to the female-stereotypical profile, was confirmed. There was a higher probability for girls to be categorized into the female-stereotypical interest profile than into all other profiles (compared to high: *b* = 2.30, SE = 0.18; low: *b* = 1.78, SE = 0.18; male: *b* = 8.93, SE = 3.65).

However, as the previous analysis showed girls were less represented in the male-stereotypical profile than boys in the female-stereotypical profile, contradicting hypothesis **H3b** that almost no boys should be in the female-stereotypical profile. Therefore, hypothesis H3b was not confirmed.

Hypothesis **H3c** was confirmed, in that there was a higher probability to be categorized into the female-stereotypical interest profile than into the low interest profile (*b* = 0.07, SE = 0.02) with higher reasoning ability.

### Hypotheses H4a, H4b, and H4c

Finally, there was a higher probability for boys to be categorized into the male interest profile than into the female interest profile (*b* = −8.93, SE = 3.65). The effect of gender, however, was insignificant at the 95% level when comparing the probability of students to belong to the low interest profile over the male interest profile (*b* = −7.15, SE = 3.64) and the probability to belong to the high interest profile over the male interest profile (*b* = −6.63, SE = 3.65). Therefore, hypothesis **H4a** that boys most likely belong to the male-stereotypical profile over all other profiles was not confirmed.

Hypothesis **H4b** had to be rejected, because there was no significant interaction effect of gender and gender-role orientation when comparing the male interest profile to any other profile (compared to high: *b* = −2.36, SE = 1.74; low: *b* = −2.66, SE = 1.74; female: *b* = −1.85, SE = 1.74). Girls who had an egalitarian gender-role orientation had no higher probability to belong to the male interest profile compared to any other profile.

Lastly, hypothesis **H4c** was confirmed; there was a higher probability to be categorized into the male interest profile than into the low interest profile (*b* = 0.15, SE = 0.03) with higher reasoning abilities.

## Discussion

This study investigated gender-stereotypical interest profiles of combined academic and vocational interests and their relationship to students’ gender-role orientation, gender, and reasoning ability in a large representative sample of sixth grade students from Germany.

The first goal was to identify different interest profiles: two undifferentiated and two gender-stereotypical profiles. Results confirmed one generally high interest profile, one generally low interest profile, one male-stereotypical interest profile, and one female-stereotypical interest profile into which students could be categorized. Gender-stereotypical interest profiles were identified by strong interest in gender-stereotypical domains and weak interest in non-stereotypical domains. The largest profile was the female-stereotypical interest profile, followed by the male-stereotypical interest profile, the high interest profile, and the low interest profile. Still 17% of all students were categorized into the low interest profile. This means that they did not show particular interest in any domain.

We had expected that more girls would be categorized into the male-stereotypical interest profile than boys into the female-stereotypical interest profile since boys face greater sanctions for associating with the lower status group (Liben et al., 2001; Rudman et al., 2012; Croft et al., 2015) and girls and women were shown to aspire to more agentic domains and leadership positions over the past decades (Eagly and Carli, 2003). This expectation was not confirmed by the current findings. In fact, the distribution was reversed: more boys were found in the female-stereotypical interest profile than girls in the male-stereotypical interest profile. Therefore, it seems that the female-stereotypical interest profile is the most desirable and more boys divert their interests to non-stereotypical domains than girls. One explanation of this unexpected finding is a positive connotation of communal tasks and domains: children of our age group consider prosocial behaviors as highly moral (e.g., Eisenberg, 2018). This might explain, why it is less threatening for boys to engage in the female-stereotyped interest domains because the tasks and behavioral descriptions are considered as prosocial behaviors (e.g., helping others to feel comfortable) and age appropriate.

In a next step, profile- and predictor-specific hypotheses were tested. The analyses revealed that, in line with previous research on the relationship between gender-role orientation and academic competence (e.g., Ehrtmann and Wolter, 2018), the probability of being categorized into the low interest profile rather than the female-stereotypical or high interest profiles increased with a traditional gender-role orientation. However, students’ gender-role orientation did not differentiate between the likelihood of being categorized into the low versus male-stereotypical interest profiles. Having an egalitarian gender-role orientation increased the probability of being categorized into the high interest profile compared to the low and male-stereotypical interest profiles, but not compared to the female-stereotypical interest profile. These results of students’ gender-role orientation suggest that increasingly egalitarian students are more likely to have high or female-stereotypical interest profiles rather than low or male-stereotypical interest profiles. Possibly, egalitarian attitudes toward gender roles indicate more liberal attitudes in general. Research on stereotypes associated with different subject domains has shown that female-connoted domains – such as languages, arts, or social activities – are more strongly associated with liberal values such as autonomy and freedom of expression, compared to mathematics, sciences, or domains dominated by manual activities (e.g., Hannover and Kessels, 2004; Makarova and Herzog, 2015). Conversely, increasingly traditional students are more likely to have either a low or male-stereotypical interest profile rather than a high or female-stereotypical interest profile.

Reasoning ability was also relevant to the probability of students’ membership in specific profiles. Students with higher reasoning ability were also more likely to have a high, female-, or male-stereotypical interest profile rather than a low interest profile. Surprisingly, high reasoning ability was also connected to a male-stereotypical interest profile compared to a high interest profile. Reasoning did not, however, differentiate between the probability of having a high or a female-stereotypical interest profile. This shows that reasoning ability differentially predicts low interests versus other profiles. It also shows that reasoning ability may be connected especially to male-stereotypical interests, i.e., mathematics, realistic, investigative, and enterprising interests. Similar results were found by Päßler et al. (2015), who reported a connection between a general intelligence factor and realistic, investigative, and conventional interests.

As expected, gender was also related to the probability of belonging to a profile. Girls more likely belonged to the female-stereotypical interest profile than to other profiles. Boys more likely belonged to the male-stereotypical interest profile than to the female-stereotypical interest profiles. Yet, the gender effect for the probability of displaying the male-stereotypical compared to the low and high interest profiles was not significant. Students’ gender did not seem to be relevant to the probability of memberships when they expressed either low or high compared to male-stereotypical interests.

Overall, these results showed that students’ interests were already rather differentiated regarding gender stereotypes in early secondary school years. This is in line with previous studies investigating gender differences in academic interests (Wigfield and Cambria, 2010) and vocational interests (von Maurice and Bäumer, 2015). Yet, unexpectedly, more girls expressed gender-stereotypical interests than boys and a considerable number of boys expressed female-stereotypical interests. Reasons may lie in the nature of the assessed interest domains: vocational female-stereotypical interests may – especially for 12-year-old children – not be associated with certain, more concrete occupations, but are rather considered as general activities that are performed and encouraged regardless of vocational aspirations or special interests, such as helping others, being neat, or drawing a picture. Male-stereotypical interests, such as working with metal or using a microscope, must be especially sought out and warrant a special interest in the activity. In addition, gender stereotypes intensify during adolescence, especially in girls, which may result in more engagement in female activities (for an overview, see Ruble et al., 2006). Evidence for the sanctioning of boys following female-stereotypical interests could, however, not be found in the current study.

Furthermore, considering students’ gender-role orientation, the results revealed that traditional students more likely expressed low interests or male-stereotypical interests rather than high interests or female-stereotypical interests. This held true for boys and girls. A possible explanation for this connection of students’ gender-role orientation and their interest profiles could lie in the broader meaning of having egalitarian or traditional attitudes toward gender roles. Endorsing egalitarian attitudes toward gender roles indicates a more liberal world view in general with more need for autonomy and the freedom to express oneself, such as in languages, arts, or social activities than endorsing traditional attitudes, which may be associated with a preference for stricter rules and highly structured environments as reflected in more clearly defined activities like mathematics, sciences, or manual activities.

### Limitations and Outlook

When considering the results, it is important to note that most variables were assessed in grade six, and therefore primarily cross-sectional data were presented in this study. For this reason, the relationships were only correlational in nature and no causal inferences should be drawn.

Reasoning ability was used in order to measure cognitive abilities. This is, of course, only one indicator of fluid cognitive abilities and might be more strongly connected to interests in mathematical skills or science than to interests in more female-connoted domains such as reading or arts. Other indicators of cognitive abilities that might tap more into other areas, such as language processing, could be investigated in further studies.

It has to be kept in mind that academic and vocational interests were assessed with different scales; therefore, the assessed concepts could also differ slightly from each other. Especially some of the subscales of the vocational interests scale showed low internal consistencies, specifically conventional interest. This might be due to the small number of items used for each interest domain (Cortina, 1993).

Furthermore, it is important to mention that correlations and odds ratios of the logistic regression analyses are mostly rather small to medium. In a large sample, such as was used in this study, even small effects become significant; this needs to be kept in mind when interpreting the results and thinking about implications.

Social desirability might have challenged the explicit assessment of students’ gender-role orientation, as this study relied on self-reported data.

In a next possible step, the change over time of these interest profiles of both academic and vocational interests and their predictive value for academic outcomes and choices in later years should be examined. It is plausible to assume that gender-stereotypical interests become less important in favor of even more differentiated interests in only specific domains. Another interesting aspect in future studies of latent interest profiles could be the inclusion of context factors, such as class or teacher stereotypes, which might influence students’ interests and choices.

Also, our analysis was clearly focused on individuals and is mute with respect to the question where girls’ and boys’ interests come from. Future studies need to broaden the perspective to include cultural explanations relating to masculinity and femininity and how the changes that have occurred over the last decades in how boys and girls are perceived impact the development of academic and vocational interests in boys and girls.

### Conclusions

Students in sixth grade already seem to have rather gender-stereotyped interests in both academic and vocational domains, which is shown by gender differences in all interest scales and the finding of two gender-stereotypical interest profiles, even though stereotypicality varied for individuals and, in some cases, was even reversed This effect is more pronounced in girls than in boys in middle childhood, which might be due to girls’ more advanced general development and an intensifying of gender stereotypes (Hill and Lynch, 1983; Ruble et al., 2006).

Furthermore, it seems that boys at this age are not as stereotyped as girls. Quite a few boys even have gender-atypical interests. The question remains why these interests are not transferred into later occupational choices, as evidenced by the low number of men in female-dominated occupations (e.g., Hausmann and Kleinert, 2014; Croft et al., 2015). It is concerning that only a very small percentage of girls was interested in male-connoted areas. This finding is repeatedly reflected in statistical data which confirm the underrepresentation of women in STEM study subjects and occupational fields (Statistisches Bundesamt [German Federal Statistical Office], 2017). Promoting girls’ interest in mathematical, practical, scientific, and entrepreneurial areas seems to be a challenge even in children as young as about 12 years old. Additionally, fostering gender-egalitarian attitudes in all children could promote higher interests in a broad range of areas.

Finally, it is alarming that 17% of students did not have any particular interests within the academic and vocational areas explored in this study. A goal in future education should certainly be to foster students’ interests at a young age in order to help them build skills and aspirations.

## Ethics Statement

The Federal Ministries of Education in Germany approved the study. Ethical standards were approved by the National Educational Panel Study. Written informed consent was given by the students and their parents in accordance with the Declaration of Helsinki. Moreover, informed consent was also given by the educational institutions to take part in the study. The consent procedure was approved by a special data protection and security officer of the National Educational Panel Study. Students (as well as all other parties) could abort their participation at any time in the study. Further approval by an ethics committee was not required according to the local and national guidelines.

## Author Contributions

All three authors contributed to the conception of the article. LE conducted the statistical analyses and drafted the article with advice from IW. IW and BH critically reviewed the article and substantially contributed to the Theoretical Background and Discussion sections.

### Conflict of Interest Statement

The authors declare that the research was conducted in the absence of any commercial or financial relationships that could be construed as a potential conflict of interest.

## Footnotes

^2^An exploratory factor analysis showed a five-factor model. Realistic and investigative interests combined accounted for one factor while all remaining factors represented one interest type each, as is expected according to the Holland model. We decided, however, to use the theoretical distinction of six factors as proposed by Holland (1997). Also, the factor analysis did not suggest merging the subscale of conventional interests, which had the lowest reliability (*α* = 0.53), with another subscale.

^3^An earlier model included parents’ socioeconomic status as a control variable. Since this variable showed a large number of missing values and excluding it did not change the results, it was excluded.

^4^The Mplus model outputs for the five- and six-class solutions can be found here: https://osf.io/wv2at/?view_only=9dcfea10b3144c0fb49f359f7aac17d9
